# Supplementary material for: Evidence of conditioned behavior in amoebae
Source: Nat Commun. 2019 Aug 15;10:3690. doi: 10.1038/s41467-019-11677-w (PMC6695432; doi:10.1038/s41467-019-11677-w)
Supplement: Supplementary file 2 — Description of Additional Supplementary Files [file 41467_2019_11677_MOESM2_ESM.pdf]

Description of Additional Supplementary Files

File Name: Supplementary Data 1
Description: Experimental set-up model. This file contains an accurate three-dimensional AutoCAD reproduction of the experimental set-up (electrophoresis blocks, agar bridges and glass structure) used in all the experiments.

File Name: Supplementary Data 2
Description: Digitized Amoeba trajectories. This file contains the corresponding Matlab files that resulted from the quantification of the videos.
