# Supplementary material for: Evidence of conditioned behavior in amoebae
Source: Nat Commun. 2019 Aug 15;10:3690. doi: 10.1038/s41467-019-11677-w (PMC6695432; doi:10.1038/s41467-019-11677-w)
Supplement: Supplementary file 5 — Reporting Summary [file 41467_2019_11677_MOESM5_ESM.pdf]

nature research

Corresponding author(s): Ildefonso M. De la Fuente

Last updated by author(s): Jul 10, 2019

Reporting Summary

Nature Research wishes to improve the reproducibility of the work that we publish. This form provides structure for consistency and transparency in reporting. For further information on Nature Research policies, see Authors & Referees and the Editorial Policy Checklist.

Statistics

For all statistical analyses, confirm that the following items are present in the figure legend, table legend, main text, or Methods section.

n/a	Confirmed	
☐	☒	The exact sample size (*n*) for each experimental group/condition, given as a discrete number and unit of measurement
☐	☒	A statement on whether measurements were taken from distinct samples or whether the same sample was measured repeatedly
☐	☒	The statistical test(s) used AND whether they are one- or two-sided<br>*Only common tests should be described solely by name; describe more complex techniques in the Methods section.*
☒	☐	A description of all covariates tested
☐	☒	A description of any assumptions or corrections, such as tests of normality and adjustment for multiple comparisons
☐	☒	A full description of the statistical parameters including central tendency (e.g. means) or other basic estimates (e.g. regression coefficient) AND variation (e.g. standard deviation) or associated estimates of uncertainty (e.g. confidence intervals)
☐	☒	For null hypothesis testing, the test statistic (e.g. *F*, *t*, *r*) with confidence intervals, effect sizes, degrees of freedom and *P* value noted<br>*Give P values as exact values whenever suitable.*
☒	☐	For Bayesian analysis, information on the choice of priors and Markov chain Monte Carlo settings
☒	☐	For hierarchical and complex designs, identification of the appropriate level for tests and full reporting of outcomes
☒	☐	Estimates of effect sizes (e.g. Cohen's *d*, Pearson's *r*), indicating how they were calculated

Our web collection on statistics for biologists contains articles on many of the points above.

Software and code

Policy information about availability of computer code

Data collection	Cell tracking was made using the TrackMate 4.0.0 open source software in ImageJ (http://fiji.sc/TrackMate).
Data analysis	Trajectories were analized by Matlab R2018a software.

For manuscripts utilizing custom algorithms or software that are central to the research but not yet described in published literature, software must be made available to editors/reviewers. We strongly encourage code deposition in a community repository (e.g. GitHub). See the Nature Research guidelines for submitting code & software for further information.

Data

Policy information about availability of data

All manuscripts must include a data availability statement. This statement should provide the following information, where applicable:

- Accession codes, unique identifiers, or web links for publicly available datasets
- A list of figures that have associated raw data
- A description of any restrictions on data availability

All original videos obtained in the experiments can be found in figshare, with doi: 10.6084/m9.figshare.8241284 (https://figshare.com/s/c59323fabced0c533fae) On the other hand, movies showing the main experimental procedures can be found in figshare with the doi: 10.6084/m9.figshare.8868326 (https://figshare.com/articles/Set-Up_Video_Files/8868326).

Field-specific reporting

Please select the one below that is the best fit for your research. If you are not sure, read the appropriate sections before making your selection.

☒ Life sciences ☐ Behavioural & social sciences ☐ Ecological, evolutionary & environmental sciences

For a reference copy of the document with all sections, see nature.com/documents/nr-reporting-summary-flat.pdf

Life sciences study design

All studies must disclose on these points even when the disclosure is negative.

Sample size	We took sample sizes big enough for applying properly the significance test (Wilcoxon ranksum test), as is indicated in Fahoome, G., & Sawilowsky, S. S. (2000). Review of Twenty Nonparametric Statistics and Their Large Sample Approximations.
Data exclusions	No data was excluded from the analysis.
Replication	We have included a detailed explanation of all the phases of the experiment, including videos of each of them, in order to facilitate reproducibility.
Randomization	The cells were randomly selected in all the cases.
Blinding	Researchers involved in the quantitative analysis of the cellular trajectories were never aware of what scenario each trajectory belonged to. Only when all the trajectories were quantified and processed, the researchers in charge of recording the amoeba's movements informed the rest of the team of which trajectories belonged to each experiment or control.

Reporting for specific materials, systems and methods

We require information from authors about some types of materials, experimental systems and methods used in many studies. Here, indicate whether each material, system or method listed is relevant to your study. If you are not sure if a list item applies to your research, read the appropriate section before selecting a response.

Materials & experimental systems

n/a	Involved in the study	
☒	☐	Antibodies
☐	☒	Eukaryotic cell lines
☒	☐	Palaeontology
☒	☐	Animals and other organisms
☒	☐	Human research participants
☒	☐	Clinical data

Methods

n/a	Involved in the study	
☒	☐	ChIP-seq
☒	☐	Flow cytometry
☒	☐	MRI-based neuroimaging

Eukaryotic cell lines

Policy information about cell lines

Cell line source(s)	Amoeba proteus: Carolina Biological Supply Company, Burlington, NC.Item # 131306. Metamoeba Leningradensis: Culture Collection of Algae and Protozoa, Oban, Scotland, UK, CCAP catalog number 1503/6.
Authentication	No authentication procedures were performed by the authors.
Mycoplasma contamination	All the relevant testing was performed either by the ATCC or the providers.
Commonly misidentified lines (See ICLAC register)	There are no commonly misidentified lines of relevance.
